# Factors Associated with Home Food Environment in Low-Income Overweight or Obese Pregnant Women

**DOI:** 10.3390/nu14040869

**Published:** 2022-02-18

**Authors:** Mei-Wei Chang, Chyongchiou J. Lin, Rebecca E. Lee, Duane T. Wegener

**Affiliations:** 1 College of Nursing, The Ohio State University, 1585 Neil Avenue, Columbus, OH 43210, USA; lin.3782@osu.edu; 2 Center for Health Promotion and Disease Prevention, Edson College of Nursing and Health Innovation, Arizona State University, Phoenix, AZ 85004, USA; releephd@yahoo.com; 3 Department of Psychology, The Ohio State University, Columbus, OH 43210, USA; wegener.1@osu.edu

**Keywords:** low-income, obesity, pregnant women, stress, ultra-processed foods

## Abstract

Limited research has examined factors associated with home food availability. This study investigated the associations among demographics, body mass index category, stress, and home food availability among low-income overweight or obese pregnant women. This cross-sectional study enrolled 332 participants who were non-Hispanic black or white. We performed logistic regression modeling for unprocessed food, processed food, overall ultra-processed food, and three subcategories of ultra-processed food (salty snacks, sweet snacks and candies, and soda). Black women were less likely than white women to have large amounts of processed foods (OR = 0.56), salty snacks (OR = 0.61), and soda (OR = 0.49) available at home. Women with at least some college education or at least a college education were more likely to have large amounts of unprocessed food (OR = 2.58, OR = 4.38 respectively) but less likely to have large amounts of soda (OR = 0.44; OR = 0.22 respectively) available at home than their counterparts. Women with higher stress were less likely to have large amounts of unprocessed food available at home (OR = 0.58) than those with lower stress. Home food availability varied by race, education, and levels of stress in low-income overweight or obese pregnant women.

## 1. Background

Ultra-processed foods are industrial processed foods that provide approximately 89% of the added sugar in the American diet [1]. Approximately 60% of the total daily calories of American adults, especially those with low incomes, come from ultra-processed foods [2]. Ultra-processed foods such as sugar-sweetened beverages, fruit drinks, cakes, cookies, pies, salty snacks, and pizza [3,4] are associated with poor dietary quality. Ultra-processed foods are low in protein, fiber, and micronutrients (e.g., vitamins A, C, and D, and potassium) but high in carbohydrates, added sugar, sodium, and saturated fat [2,5].

High consumption of ultra-processed foods is harmful to women during pregnancy and after delivery. American pregnant women consume a high percentage of their calories [6] (approximately 50%) from ultra-processed foods [7]. Evidence has shown a link between consumption of ultra-processed foods and excessive gestational weight gain [8], prevalent in low-income overweight or obese pregnant women [9,10]. A 1% increase in energy intake from ultra-processed foods has been associated with a nearly 3-pound increase in gestational weight gain [8]. Excessive gestational weight gain is associated with adverse maternal outcomes, such as gestational diabetes and gestational hypertension [11,12], both of which increase women’s risk for type 2 diabetes [13] and hypertension [14] later in life. These chronic conditions are associated with ultra-processed food intake [4,15].

Excessive gestational weight gain is preventable through healthy eating, such as increasing fruit and vegetable intake and reducing ultra-processed food intake [16]. As food consumption has been associated with the food that is available at home [17], one possible strategy to prevent excessive gestational weight gain in low-income overweight or obese pregnant women is to promote the storage of healthy foods at home. Lower availability of high-fat foods at home has been associated with reduced caloric intake [18] and weight loss [18,19,20]. On the contrary, unhealthy food availability at home, such as storing a high number of ultra-processed food items, has been associated with poorer diet quality [21,22,23], high caloric intake, less fruit and vegetable consumption, unhealthy food preparation [24], and eating less healthy family dinners [25].

The home food environment, hereafter food availability at home, is a modifiable risk factor for excessive gestational weight gain and chronic health conditions. To improve maternal health outcomes, it is imperative to identify potential factors affecting home food availability for pregnant women, especially those with low incomes and with pre-pregnancy overweight or obesity. However, limited research has been conducted in this area. Available data have shown that non-Hispanic blacks are more likely to consume ultra-processed foods [2] and added sugar than non-Hispanic whites [6]. Mothers with less formal education were less likely to store fruits [26] but were more likely to store ultra-processed foods at home [2]. Full-time employed mothers were less likely to prepare meals at home than the part-time and unemployed mothers [27]. Additionally, fewer fruits and vegetables and more high-fat snacks available at home are linked to obesity [28,29]. Moreover, higher levels of stress, which are highly prevalent in low-income overweight or obese pregnant women [30], have been associated with less healthy food being available at home [27,31]. However, it is unclear whether the previous findings could be generalized to low-income overweight or obese pregnant women. The objective of this secondary analysis was to investigate the associations in this priority population of demographics (race, education, employment, trimester, and smoking), body mass index (BMI) category, and stress, with each of the six food categories available at home: unprocessed foods, processed foods, overall ultra-processed foods, and three subcategories of ultra-processed foods (salty snacks, sweet snacks and candies, and soda). We hypothesize that these independent variables are associated with food categories available at home.

## 2. Subjects and Method

### 2.1. Setting and Participants

Participants were recruited from the Special Supplemental Nutrition Program for Women, Infants, and Children (WIC) in Michigan, USA. WIC is a federally funded program that provides services, such as nutrition counseling, referrals, and food vouchers, to low-income (defined as <185% of the federal poverty line) pregnant, postpartum, and breastfeeding women and children under 5 years old. To be eligible to participate in the study, pregnant women had to be enrolled in WIC, be 18 years or older, be non-Hispanic black or white (hereafter, black and white), and have pre-pregnancy BMI of at least 25.0 kg/m^2^ (calculated using self-reported height and weight). Detailed descriptions of the recruitment have been published elsewhere [32]. Eligible women (N = 332) each provided a written consent form prior to participating in this cross-sectional study. The study was conducted in accordance with the Declaration of Helsinki, and the procedure was approved by Institutional Review Board at Michigan State University.

### 2.2. Measures

Participants responded to survey questions via a self-administered pencil-and-paper survey while they waited for their WIC appointments.

### 2.3. Demographics

Participants self-reported age, race/ethnicity, education, employment, and smoking status. Each also reported the date of her last menstrual cycle, which was used to calculate gestational age and trimester status.

### 2.4. Perceived Stress, Hereafter Stress

Stress was measured using the Perceived Stress Scale (9 items) with reported validity and reliability [33]. The survey measures the degree to which situations in one’s life are appraised as stressful. Responses ranged from rarely or never (1) to usually or always (4). The overall stress score that ranged from 4 to 36 was the sum of the 9 items with higher scores indicating higher levels of stress.

### 2.5. Home Food Environment

The Healthy Home Food Survey was used to measure home food environment or food item availability at home [34]. The survey has shown acceptable feasibility, reliability, and validity [34]. Participants responded (yes = 1, no = 0) as to whether to food items were available at home that day. We applied the NOVA, a food classification system, to categorize food items. NOVA food categories, organized according to the extent of the processing they undergo, include unprocessed foods (e.g., fresh, dried, and frozen fruits, and fresh and frozen vegetables), processed foods (e.g., canned fruits and vegetables), and ultra-processed foods (salty snacks, sweet snacks, candies, and soda) [3]. The unprocessed foods had 74 items: 24 fresh fruits (e.g., apples, bananas, oranges), 10 dried fruits (e.g., apples, apricots), 8 frozen fruits (e.g., apples, blueberries), 24 fresh vegetables (e.g., broccoli, cabbage), and 8 frozen vegetables (e.g., broccoli, mixed vegetables). Processed foods had 24 items: 14 canned or jarred fruits (e.g., applesauce, peaches) and 10 canned or jarred vegetables (e.g., green beans, corn). Ultra-processed foods (28 items) included 3 subcategories: 3 salty snacks (e.g., corn/tortilla chips, potato chips), 6 sweet snacks (e.g., cake, cookies), 8 candies (e.g., chocolate bars, caramel, hard candy), and 11 sodas (e.g., Coca-Cola, Dr. Pepper). We summed the foods within each of the 3 food categories (unprocessed foods ranged from 0 to 74, processed foods ranged from 0 to 24, overall ultra-processed foods ranged from 0 to 28) and within each ultra-processed food subcategory (salty snacks ranged from 0 to 3, sweet snacks and candies ranged from 0 to 14, and soda ranged from 0 to 11). For all scores created, the higher the score, the more food items available at home.

### 2.6. Statistical Analysis

The study hypothesis was specified prior to data collection. Additionally, the analytic plan was pre-specified. Descriptive analyses were performed for demographics, BMI category, and stress measures. Logistic regression modeling was conducted to investigate the associations among demographics, BMI category, stress, and food category items available at home among low-income overweight or obese pregnant women. The outcome or dependent variables included 6 food categories: unprocessed foods, processed foods, overall ultra-processed foods, and 3 subcategories of ultra-processed foods (salty snacks, sweet snacks and candies, and soda). As Kolmogorov–Smirnov test results showed that all outcome variables were not normally distributed, a median cutoff value was used to dichotomize each outcome variable (≤median = a low number of food items available at home and >median = a high number of food items available at home). Independent variables were race (black vs. white), education (high school graduate, some college, or college graduate and higher vs. less than high school), employment status (employed vs. unemployed), trimester (second or third trimester vs. the first trimester), smoking (smoker vs. non-smoker), BMI category (obesity vs. overweight), and stress (higher vs. lower levels of stress). Statistical significance was set to *p* < 0.05 for all tests. Statistical software SAS (version 9.4) was used for all analytical procedures (SAS Institute, Inc., Cary, NC, USA).

## 3. Results

Table 1 presents demographic characteristics, BMI category, and stress scores. Table 2 shows the median of each food category/subcategory at home, which were used to define the outcome values for the food categories. Table 3 shows odds ratios (OR) and 95% confidence intervals (CI) of logistic regressions for the food categories.

### 3.1. Unprocessed Food Items: Fresh, Dried and Frozen Fruits and Fresh and Frozen Vegetables

Pregnant women with some college education (OR = 2.58; 95% CI: 1.26–5.31) or college and higher education (OR = 4.38; 95% CI: 1.62–11.83) were more likely to have a high number of unprocessed food items available at home than women with less than a high school education. Women with higher levels of stress were less likely to have a high number of unprocessed food items available at home (OR = 0.58; 95% CI = 0.37–0.92) than women with lower levels of stress. Race, employment, trimester, smoking, and BMI categories were not associated with having a high number of unprocessed food items available at home.

### 3.2. Processed Food Items: Canned or Jarred Fruits and Vegetables

Black pregnant women were less likely than white pregnant women to have a high number of processed food items available at home (OR = 0.56; 95% CI: 0.34–0.90). Compared to women with less than a high school education, pregnant women with college and higher education (OR = 2.92; 95% CI: 1.10–7.76) were more likely to have a high number of processed food items available at home. Employment, trimester, smoking, BMI category, and stress were not associated with having a high number of processed food items available at home.

### 3.3. Ultra-Processed Food Items Overall: Salty Snacks, Sweet Snacks and Candies, and Soda

There were no associations between race, education, employment, smoking, or trimester and having a high number of overall ultra-processed food items available at home. Similarly, BMI category and levels of stress were not associated with having a high or low number of ultra-processed food items available at home.

### 3.4. Ultra-Processed Food Items: Salty Snacks

Black pregnant women were less likely than white pregnant women to have a high number of salty snack items available at home (OR = 0.61; 95% CI: 0.37–0.98). No associations were found between education, employment, trimester, smoker, BMI category, or stress and having a higher or lower number of salty snack items available at home.

### 3.5. Ultra-Processed Food Items: Sweet Snacks and Candies

There were no associations between race, education, employment, smoking, trimester, BMI category, or level of stress and having a higher or lower number of salty snack and candy items available at home.

### 3.6. Ultra-Processed Food Items: Soda

Low-income black pregnant women were less likely than their counterparts to have a high number of soda items available at home (OR = 0.49; 95% CI: 0.30–0.79). Compared to those with less than high school education, pregnant women with some college education (OR = 0.44; 95% CI: 0.22–0.88) or undergraduate degrees and higher (OR = 0.22; 95% CI: 0.08–0.59) were less likely to have a high number of soda items available at home. However, number of soda items available at home was not associated with stress.

## 4. Discussion

Food availability at home has been linked with dietary intake, which is associated with gestational hypertension [35,36] and gestational weight gain, especially for women with pre-pregnancy overweight or obesity [37]. The present study was the first to investigate the associations among demographics, BMI category, stress, and food category (unprocessed, processed, and ultra-processed foods) available at home in low-income overweight or obese pregnant women.

The results of the present study showed that black pregnant women were less likely (0.39 to 0.51 times) than white pregnant women to report having high numbers of processed food items, salty snack and candy items, and soda items available at home. The differences between black and white women might have related to cultural food preferences, and preparation and purchasing behavior [38,39]. Previous research has reported that home food environment is positively associated with dietary habits, and black women were more likely to have higher consumption of ultra-processed foods than white women [2,6]. As we are the first group to make such a comparison, these findings are challenging to interpret. Perhaps low-income black pregnant women have less variety in the food they store at home than their white counter parts, relying more on fast food restaurants and convenience stores more commonly located in low-income neighborhoods with higher proportions of black residents [40,41].

As the present study did not examine the association between home food availability and dietary intake, we do not know whether availability of ultra-processed food at home would be associated with poor dietary intake in black women, as suggested in previous research. Future studies may consider investigating the associations among home food availability, ultra-processed food consumption, and sources of ultra-processed food purchases (such as fast-food restaurants or corner stores).

Educational attainment seemed to account for much of the home food availability in the present sample, particularly in regard to unprocessed foods, processed foods, and soda. Women with at least some college education were 2.58–4.38 times more likely to have a high number of unprocessed food items available at home and 0.56 to 0.78 times less likely to have a high number of soda items available at home compared to women with less than a high school education, after accounting for race. Our findings are somewhat consistent with previous findings suggesting a healthier home food environment among those with higher educational attainment [2,26]. At the same time, women with at least a college education were 2.92 times more likely to have a high number of processed food items at home than women with less than a high school education, suggesting that these relations may be more complex. Having some quantity of processed foods (such as canned/jarred vegetables) might actually contribute to improved overall dietary quality, allowing quicker preparation of healthy dishes and more reliable food storage (e.g., canned vegetables readily available for an easy side-dish). We did not find that other demographic characteristics (employment, trimester, and smoking) or BMI categories were related to the likelihood of having a high number of any food group items available at home. The lack of association between BMI categories and food items available at home might have related to the present study only including overweight and obese women.

We found that women with higher levels of stress were 0.42 times less likely to have a high number of unprocessed food (fresh fruits and vegetables) items available at home. Higher levels of stress have been previously linked with food insecurity, which affects fresh fruit and vegetable available at home, leading to lower consumption of fruit and vegetable intake in low-income pregnant women [42]. Thus, screening low-income overweight or obese women with higher levels of stress during pregnancy, which is not typically performed in clinical settings or community programs serving this priority population, is important. Stress and nutrition are both particularly critical factors for fetal development during pregnancy; thus, future studies are needed to learn how to insure adequate healthful food availability in the home.

## 5. Strengths

The present study evaluated factors affecting the number of food items available at home by asking low-income overweight or obese pregnant women to respond to a comprehensive list of food categories. We applied NOVA, a new food classification system, to classify foods into three categories: unprocessed, processed, and ultra-processed foods.

## 6. Limitations

There are limitations of this study. The analyses were performed using cross-sectional data. Thus, causal relationships could not be established. Rather than counting actual food items, the present study utilized self-reported current food items available at home. To reduce participants’ burden in answering the questionnaire, the study did not ask for the number of each food item available at home. Additionally, we did not assess the number of people living in the household, which might have affected the food items available at home. Moreover, we did not collect data on whether the food was eaten only by the pregnant women in the study or by other household members. Furthermore, interpretation of the associations between race and food environments must be undertaken with caution because we did not stratify race by education due to the sample size. Results of the present study might not be generalizable to low-income overweight or obese pregnant women in other geographical locations. Finally, we did not evaluate the associations between food items available at home and dietary intake behaviors.

## 7. Conclusions

Black race was associated with fewer processed foods and fewer of some ultra-processed foods (salty snacks and candies, and soda) available at home. Educational attainment may provide a nutrition buffer in some cases, as higher education was associated with higher numbers of unprocessed and processed food items available at home, and fewer soda items available at home. Additionally, higher levels of stress were negatively associated the number of unprocessed food items available at home. Longitudinal studies are needed to determine whether changes in the availability of food items at home (via direct observation instead of self-reporting) would change dietary intake behaviors. It would also be useful to investigate whether food items available at home mediate associations among demographics, BMI category, or stress, and dietary eating behaviors. Moreover, prospective studies investigating the associations between home food environment and adverse maternal and birth health outcomes are needed to potentially provide evidence that can guide the formulation of health policies. Furthermore, lifestyle interventions should perhaps focus on the home food environment and examining its impact on health outcomes.

## Figures and Tables

**Table 1:** Demographics of low-income overweight or obese pregnant women (N = 332).

Demographics		
Continuous Variable	**Mean (SD)**	**Range**
Age (years)	25.7 (5.5)	18–46
Gestational weeks	19.5 (9.9)	3–39
Body mass index (BMI)	32.5 (6.2)	25.0–60.3
Total stress score	20.4 (3.0)	12–30
Categorical Variable	**N**	**%**
Race		
Non-Hispanic White	192	57.8
Non-Hispanic Black	140	42.2
Education		
Less than high school	51	15.4
High school graduate	80	24.1
Some college	168	50.6
College graduate and higher	33	9.9
Employment Status		
Unemployed	215	64.8
Employed	117	35.2
Trimester Status		
First trimester: ≤12 weeks or less	105	31.6
Second trimester: 13–27 weeks	132	39.8
Third trimester: ≥28 weeks	95	28.6
Smoker		
Non-smoker	284	85.5
Smoker	48	14.5
BMI category		
Overweight (BMI: 25.0–29.9 kg/m^2^)	137	41.3
Obese (BMI ≥ 30.0 kg/m^2^)	195	58.7
Stress		
Lower stress (score ≤20)	176	53.0
Higher stress (score >20)	156	47.0

**Table 2:** Food items available at home for low-income overweight or obese pregnant women (N = 332).

	Mean	SD	Median	Min	Max
Unprocessed foods	11.3	7.8	10	0	52
Processed foods	5.0	3.3	5	0	18
Ultra-processed foods: Overall	3.7	3.1	3	0	21
Ultra-processed foods: Salty Snacks	1.2	1.0	1	0	3
Ultra-processed foods: Sweet Snacks and Candies	2.6	2.3	2	0	12
Ultra-processed foods: Soda	1.0	1.4	0	0	10

Unprocessed foods = fresh, dried, and frozen fruits; and fresh and frozen vegetables. Processed foods = canned or jarred fruit and vegetables. Ultra-processed foods: overall = salty snacks, sweet snacks and candies, and soda. Each median value presented here was used to dichotomize each outcome variable.

**Table 3:** Estimates of logistic regression by food items available at home (N = 332).

	Unprocessed Food	Processed Food	Ultra-Processed Foods			
			Overall	Salty Snacks	Sweet Snacks and Candy	Soda
Independent Variable	Odds Ratio (95% CI)	Odds Ratio (95% CI)	Odds Ratio (95% CI)	Odds Ratio (95% CI)	Odds Ratio (95% CI)	Odds Ratio (95% CI)
Race (ref: White)						
Black	1.60 (0.99–2.59)	0.56 (0.34–0.90) *	1.06 (0.66–1.69)	0.61 (0.37–0.98) *	1.08 (0.67–1.74)	0.49 (0.30–0.79) *
Education (ref: <high school)						
High school graduate	1.97 (0.90–4.31)	1.82 (0.82–4.01)	0.86 (0.41–1.80)	1.41 (0.66–3.05)	0.63 (0.30–1.32)	0.84 (0.39–1.81)
Some college	2.58 (1.26–5.31) *	1.53 (0.74–3.18)	0.68 (0.34–1.33)	0.91 (0.44–1.86)	0.52 (0.27–1.04)	0.44 (0.22–0.88) *
College and higher	4.38 (1.62–11.83) *	2.92 (1.10–7.76) *	0.74 (0.29–1.87)	1.40 (0.53–3.65)	1.40 (0.54–3.59)	0.22 (0.08–0.59) *
Employment (ref: unemployed)						
Employed	1.03 (0.64–1.67)	1.12 (0.69–1.82)	0.90 (0.56–1.46)	1.16 (0.71–1.90)	0.98 (0.60–1.59)	0.95 (0.58–1.54)
Trimester (ref: ≤12 weeks)						
13–27 weeks	0.87 (0.51–1.50)	0.89 (0.51–1.53)	1.12 (0.65–1.91)	1.46 (0.83–2.55)	1.06 (0.62–1.82)	1.51 (0.87–2.62)
≥28 weeks	0.79 (0.44–1.43)	1.20 (0.67–2.17)	1.28 (0.72–2.28)	1.52 (0.83–2.80)	0.90 (0.50–1.63)	1.61 (0.89–2.90)
Smoking (ref: non-smoker)						
Smoker	1.97 (0.99–3.91)	1.65 (0.85–3.21)	0.98 (0.51–1.90)	1.35 (0.69–2.63)	1.14 (0.59–2.23)	0.53 (0.27–1.05)
BMI category (ref: overweight)						
Obesity	0.64 (0.40–1.01)	0.69 (0.43–1.10)	0.80 (0.51–1.25)	0.80 (0.50–1.21)	0.70 (0.45–1.11)	0.93 (0.59–1.48)
Stress (ref: lower stress)						
Higher stress	0.58 (0.37–0.92) *	0.84 (0.53–1.33)	0.79 (0.51–1.24)	0.93 (0.59–1.46)	0.69 (0.44–1.10)	0.96 (0.60–1.52)

≤12 weeks = first trimester, 13–27 weeks = second trimester, ≥28 weeks = third trimester. Unprocessed foods = fresh, dried, and frozen fruits; and fresh and frozen vegetables. Processed foods = canned or jarred fruit and vegetables. Ultra-processed foods = salty snacks, sweet snacks and candy, and soda. * *p* < 0.05; CI: confidence interval.

## Data Availability

No applicable.

## References

[1] Juul F., Martinez-Steele E., Parekh N., Monteiro C.A., Chang V.W. (2018). Ultra-processed food consumption and excess weight among US adults. Br. J. Nutr..

[2] Baraldi L.G., Martinez Steele E., Canella D.S., Monteiro C.A. (2018). Consumption of ultra-processed foods and associated sociodemographic factors in the USA between 2007 and 2012: Evidence from a nationally representative cross-sectional study. BMJ Open.

[3] Martinez Steele E., Baraldi L.G., Louzada M.L., Moubarac J.C., Mozaffarian D., Monteiro C.A. (2016). Ultra-processed foods and added sugars in the US diet: Evidence from a nationally representative cross-sectional study. BMJ Open.

[4] Yang Q., Zhang Z., Gregg E.W., Flanders W.D., Merritt R., Hu F.B. (2014). Added sugar intake and cardiovascular diseases mortality among US adults. JAMA Intern. Med..

[5] Rauber F., da Costa Louzada M.L., Steele E.M., Millett C., Monteiro C.A., Levy R.B. (2018). Ultra-Processed Food Consumption and Chronic Non-Communicable Diseases-Related Dietary Nutrient Profile in the UK (2008–2014). Nutrients.

[6] Ervin R.B., Ogden C.L. (2013). Consumption of Added Sugars among U.S. Adults, 2005–2010.

[7] Cioffi C.E., Figueroa J., Welsh J.A. (2018). Added Sugar Intake among Pregnant Women in the United States: National Health and Nutrition Examination Survey 2003–2012. J. Acad. Nutr. Diet.

[8] Rohatgi K.W., Tinius R.A., Cade W.T., Steele E.M., Cahill A.G., Parra D.C. (2017). Relationships between consumption of ultra-processed foods, gestational weight gain and neonatal outcomes in a sample of US pregnant women. PeerJ.

[9] Endres L.K., Straub H., McKinney C., Plunkett B., Minkovitz C.S., Schetter C.D., Ramey S., Wang C., Hobel C., Raju T. (2015). Postpartum weight retention risk factors and relationship to obesity at 1 year. Obstet. Gynecol..

[10] Stotland N.E., Gilbert P., Bogetz A., Harper C.C., Abrams B., Gerbert B. (2010). Preventing Excessive Weight Gain in Pregnancy: How Do Prenatal Care Providers Approach Counseling?. J. Womens Health.

[11] Heslehurst N., Simpson H., Ells L.J., Rankin J., Wilkinson J., Lang R., Brown T.J., Summerbell C.D. (2008). The impact of maternal BMI status on pregnancy outcomes with immediate short-term obstetric resource implications: A meta-analysis. Obes. Rev..

[12] Koebnick C., Smith N., Huang K., Martinez M.P., Clancy H.A., Kushi L.H. (2012). The prevalence of obesity and obesity-related health conditions in a large, multiethnic cohort of young adults in California. Ann. Epidemiol..

[13] Casagrande S.S., Linder B., Cowie C.C. (2018). Prevalence of gestational diabetes and subsequent Type 2 diabetes among U.S. women. Diabetes Res. Clin. Pract..

[14] Riise H.K.R., Sulo G., Tell G.S., Igland J., Nygård O., Iversen A., Daltveit A.K. (2018). Association Between Gestational Hypertension and Risk of Cardiovascular Disease Among 617 589 Norwegian Women. J. Am. Heart Assoc..

[15] Brown I.J., Stamler J., Van Horn L., Robertson C.E., Chan Q., Dyer A.R., Huang C.C., Rodriguez B.L., Zhao L., Daviglus M.L. (2011). Sugar-sweetened beverage, sugar intake of individuals, and their blood pressure: International study of macro/micronutrients and blood pressure. Hypertension.

[16] Shieh C., Cullen D.L., Pike C., Pressler S.J. (2018). Intervention strategies for preventing excessive gestational weight gain: Systematic review and meta-analysis. Obes. Rev..

[17] Ledoux T.A., Mama S.K., O′Connor D.P., Adamus H., Fraser M.L., Lee R.E. (2012). Home Availability and the Impact of Weekly Stressful Events Are Associated with Fruit and Vegetable Intake among African American and Hispanic/Latina Women. J. Obes..

[18] Gorin A.A., Wing R.R., Fava J.L., Jakicic J.M., Jeffery R., West D.S., Brelje K., Dilillo V.G. (2008). Look AHERG: Weight loss treatment influences untreated spouses and the home environment: Evidence of a ripple effect. Int. J. Obes..

[19] Krukowski R.A., Harvey-Berino J., West D.S. (2010). Differences in home food availability of high- and low-fat foods after a behavioral weight control program are regional not racial. Int. J. Behav. Nutr. Phys. Act.

[20] Gorin A.A., Raynor H.A., Fava J., Maguire K., Robichaud E., Trautvetter J., Crane M., Wing R.R. (2013). Randomized controlled trial of a comprehensive home environment-focused weight-loss program for adults. Health Psychol..

[21] Fulkerson J.A., Nelson M.C., Lytle L., Moe S., Heitzler C., Pasch K.E. (2008). The validation of a home food inventory. Int. J. Behav. Nutr. Phys. Act..

[22] Raynor H.A., Polley B.A., Wing R.R., Jeffery R.W. (2004). Is dietary fat intake related to liking or household availability of high- and low-fat foods?. Obes. Res..

[23] Trapp G.S., Hickling S., Christian H.E., Bull F., Timperio A.F., Boruff B., Shrestha D., Giles-Corti B. (2015). Individual, Social, and Environmental Correlates of Healthy and Unhealthy Eating. Health Educ. Behav..

[24] Ransley J.K., Donnelly J.K., Botham H., Khara T.N., Greenwood D.C., Cade J.E. (2003). Use of supermarket receipts to estimate energy and fat content of food purchased by lean and overweight families. Appetite.

[25] Trofholz A.C., Tate A.D., Draxten M.L., Neumark-Sztainer D., Berge J.M. (2016). Home food environment factors associated with the presence of fruit and vegetables at dinner: A direct observational study. Appetite.

[26] MacFarlane A., Crawford D., Ball K., Savige G., Worsley A. (2007). Adolescent home food environments and socioeconomic position. Asia Pac. J. Clin. Nutr..

[27] Bauer K.W., Hearst M.O., Escoto K., Berge J.M., Neumark-Sztainer D. (2012). Parental employment and work-family stress: Associations with family food environments. Soc. Sci. Med..

[28] Gorin A.A., Phelan S., Raynor H., Wing R.R. (2011). Home food and exercise environments of normal-weight and overweight adults. Am. J. Health Behav..

[29] Porter H., West D.S., Cleves M.A., Saylors M.E., Andres A., Krukowski R.A. (2018). Association Between Household Food Environment and Excessive Gestational Weight Gain. J. Womens Health.

[30] Chang M., Tan A., Schaffir J. (2019). Relationships between stress, demographics and dietary intake behaviours among low-income pregnant women with overweight or obesity. Public Health Nutr..

[31] Fulkerson J.A., Telke S., Larson N., Berge J., Sherwood N.E., Neumark-Sztainer D. (2019). A healthful home food environment: Is it possible amidst household chaos and parental stress?. Appetite.

[32] Chang M.W., Brown R., Nitzke S., Smith B., Eghtedary K. (2015). Stress, sleep, depression and dietary intakes among low-income overweight and obese pregnant women. Matern. Child Health J..

[33] Cohen S., Kamarck T., Mermelstein R. (1983). A global measure of perceived stress. J. Health Soc. Behav..

[34] Bryant M.J., Ward D.S., Hales D., Vaughn A., Tabak R.G., Stevens J. (2008). Reliability and validity of the Healthy Home Survey: A tool to measure factors within homes hypothesized to relate to overweight in children. Int. J. Behav. Nutr. Phys. Act..

[35] Schoenaker D.A., Soedamah-Muthu S.S., Mishra G.D. (2014). The association between dietary factors and gestational hypertension and pre-eclampsia: A systematic review and meta-analysis of observational studies. BMC Med..

[36] Schoenaker D.A., Soedamah-Muthu S.S., Callaway L.K., Mishra G.D. (2015). Prepregnancy dietary patterns and risk of developing hypertensive disorders of pregnancy: Results from the Australian Longitudinal Study on Women′s Health. Am. J. Clin. Nutr..

[37] Shen M., Smith G.N., Rodger M., White R.R., Walker M.C., Wen S.W. (2017). Comparison of risk factors and outcomes of gestational hypertension and pre-eclampsia. PLoS ONE.

[38] Cullen K., Baranowski T., Watson K., Nicklas T., Fisher J., O′Donnell S., Baranowski J., Islam N., Missaghian M. (2007). Food category purchases vary by household education and race/ethnicity: Results from grocery receipts. J. Am. Diet Assoc..

[39] Dettwyer K.A. (1989). Styles of infant feeding: Parental/caretake control of food consumption in young children. Am. Anthropol..

[40] James P., Arcaya M.C., Parker D.M., Tucker-Seeley R.D., Subramanian S.V. (2014). Do minority and poor neighborhoods have higher access to fast-food restaurants in the United States?. Health Place.

[41] Garcia M.T., Sato P.M., Trude A.C.B., Eckmann T., Steeves E.T.A., Hurley K.M., Bogus C.M., Gittelsohn J. (2018). Factors Associated with Home Meal Preparation and Fast-Food Sources Use among Low-Income Urban African American Adults. Ecol. Food Nutr..

[42] Nunnery D.L., Labban J.D., Dharod J.M. (2018). Interrelationship between food security status, home availability of variety of fruits and vegetables and their dietary intake among low-income pregnant women. Public Health Nutr..

